# Investigation of the Interaction of Ionic Surfactants with Epoxy-Based Hydrogels by SANS

**DOI:** 10.1021/acs.langmuir.4c04062

**Published:** 2025-02-06

**Authors:** Ivan Krakovský, Timur V. Tropin, Oleksandr Igorovych Ivankov, Viktor Petrenko

**Affiliations:** † Department of Macromolecular Physics, Faculty of Mathematics and Physics, Charles University, V Holešovičkách 2, 180 00 Praha 8, Czech Republic; ‡ Frank Laboratory of Neutron Physics, Joint Institute for Nuclear Research, Joliot-Curie 6, 141980 Dubna, Russia; § Basque Center for Materials, Applications & Nanostructures, Bld. Martina Casiano, UPV/EHU Science Park, Barrio Sarriena s/n, 48940 Leioa, Spain; 4 IKERBASQUE, Basque Foundation for Science, 48009 Bilbao, Spain

## Abstract

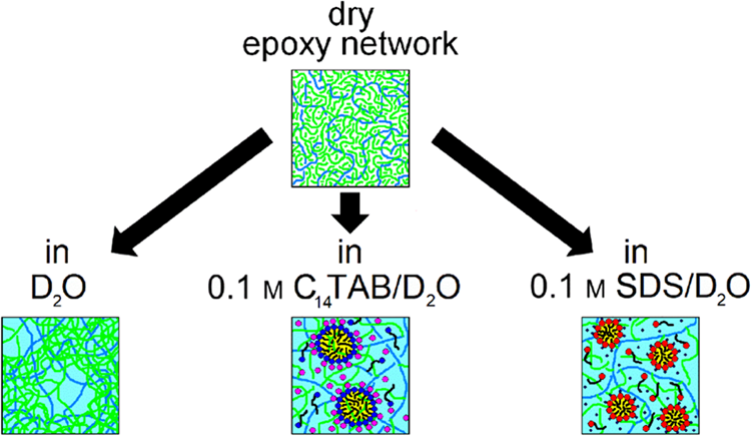

We investigated the effect of two ionic surfactants on the composition and structure of hydrogels obtained by swelling an epoxy amphiphilic polymer network (APN) using a combination of gravimetry and small-angle neutron scattering (SANS). Stoichiometric epoxy APN was synthesized by the reaction of diamino and diepoxy-terminated polypropylene (POP) and polyoxyethylene (POE). Sub- and supercritical solutions of surfactants with either cationic (myristyltrimethylammonium bromide (C_14_TAB)) or anionic (sodium dodecyl sulfate (SDS)) headgroups in heavy water were used in the preparation of the hydrogels. At supercritical concentrations, SDS exhibited a much stronger effect on the composition and structure of the hydrogels than C_14_TAB. The details of the hydrogel structure were deduced by general analysis and fitting of the experimental SANS profiles to two model scattering functions exploiting the Percus–Yevick hard-sphere (HS) model and rescaled mean spherical approximation (RMSA), respectively. In the former model, valid for the hydrogels prepared in low concentrated surfactant solutions, spherical domains of average radius of ca. 39–45 Å from a highly swollen network dispersed in a matrix of a poorly swollen network mixed with the bound alkyl surfactant tails were revealed. In the latter model, hydrogels prepared in surfactant solutions of sufficiently high concentrations consist of surfactant micelles dispersed in a matrix of a highly swollen network. The average radii of the C_14_TAB and SDS micelles formed in the hydrogels were ca. 28 and 17 Å, respectively. The strong binding of surfactant tails with POP chains and dragging of large amounts of highly mobile counterions inside the hydrogels were responsible for the effects observed. This study provides important information about the surfactant organization inside polymer hydrogels, which is important for their applications.

## Introduction

Macroscopically, polymer hydrogels are soft solids with high water content. At the microscopic level, in polymer hydrogels, a complex fluctuating three-dimensional (3D) network of interconnected polymer chains is expanded by highly mobile water molecules dispersed inside the network. Hydrogels are open systems that exchange mass and energy with the surroundings. When the hydrogel volume changes, the connectivity and topology of the polymer network must be conserved.

Overall, hydrogels have attracted much attention in modern technologies due to their potential applications as sensors, soft actuators,^1−4^ and biomedicines.^5,6^ This is based on their high sensitivity to external stimuli (e.g., change of temperature,^7^ pressure,^8,9^ solvent composition,^10,11^ pH,^12,13^ and ionic strength^14^). This responsiveness to various external changes makes hydrogels dynamic materials, allowing changes in their properties in response to specific triggers.

Systematic investigation of polymer hydrogels started in the 1950s^15−17^ with a focus on their fundamental properties. The theoretical prediction of the volume phase transition in polymer hydrogels^18^ and its first experimental observation^19^ significantly accelerated their research in the following years. So far, less attention has been paid to the effect of surfactants on the swelling behavior of hydrogels.^20−26^ However, more knowledge in this area could offer new insights into the development and advancement of their applications.

Polymer networks with different chemical structures can be found in hydrogels. In recent years, amphiphilic polymer (co)networks (APNs) have attracted attention in polymer materials science and advanced technologies.^27^ APNs are polymer networks comprising both hydrophobic and hydrophilic polymer blocks.^28^ Assembling these antagonistic blocks in the polymer network gives rise to hydrogels with a self-organized nanophase-separated structure. Hydrophobic blocks can solubilize hydrophobic compounds present in water; therefore, APNs can be used to clean water contaminated by oil products. Conversely, APNs can be exploited for the controlled release of hydrophobic compounds in industrial and medical applications.^28^ Well-defined APNs can be prepared by end-linking alternating reactions of reactive star-like hydrophobic and hydrophilic polymers.^29^ However, the synthesis of such star-like polymers is rather expensive for large-scale applications. Therefore, in our previous research,^30−35^ we prepared APNs via the end-linking reaction of commercially available telechelic polymers and used them for the production of epoxy resins. However, the topology of epoxy networks is different from that of APNs prepared from star-like polymers: the diepoxy-terminated blocks are connected to long chains to which the diamine-terminated chains are grafted by one or both terminals (see, e.g., Figure S1a in the Supporting Information).

It is well known that block copolymers of polyoxyethylene (POE) and polyoxypropylene (POP) of various molecular architectures (pluronics, synperonics, etc.) give rise to the formation of a rich variety of thermosensitive nanoassembled structures.^36,37^ In our research on epoxy APNs,^30−35^ we exploited reactive telechelic polyoxyethylene (POE) and polyoxypropylene (POP) as hydrophilic and hydrophobic blocks. Reactive star-like POE and POP dissolved in water were also used in the preparation of APNs in hydrogels, e.g., by Hiroi et al.^38^ and Mortensen and Annaka.^39^

Investigation of the structure of polymer networks swollen in good solvents by small-angle neutron scattering (SANS) revealed the presence of static (frozen) and dynamic heterogeneities, respectively, originating from the spatial distribution of network junctions and thermal movement of polymer segments.^40^ On the other hand, in the hydrogels obtained by swelling of APNs in water, the contributions of the above-mentioned heterogeneities to SANS might be superimposed by contributions from the heterogeneities arising from the nanophase separation of the blocks into water-rich (hydrophilic) and water-poor (hydrophobic) domains of a few nanometers in size (nanodomains). Nanophase separation is manifested by a distinct scattering peak at a position corresponding to hundreds of reciprocal angstroms.^30−35^

**Figure 1:**
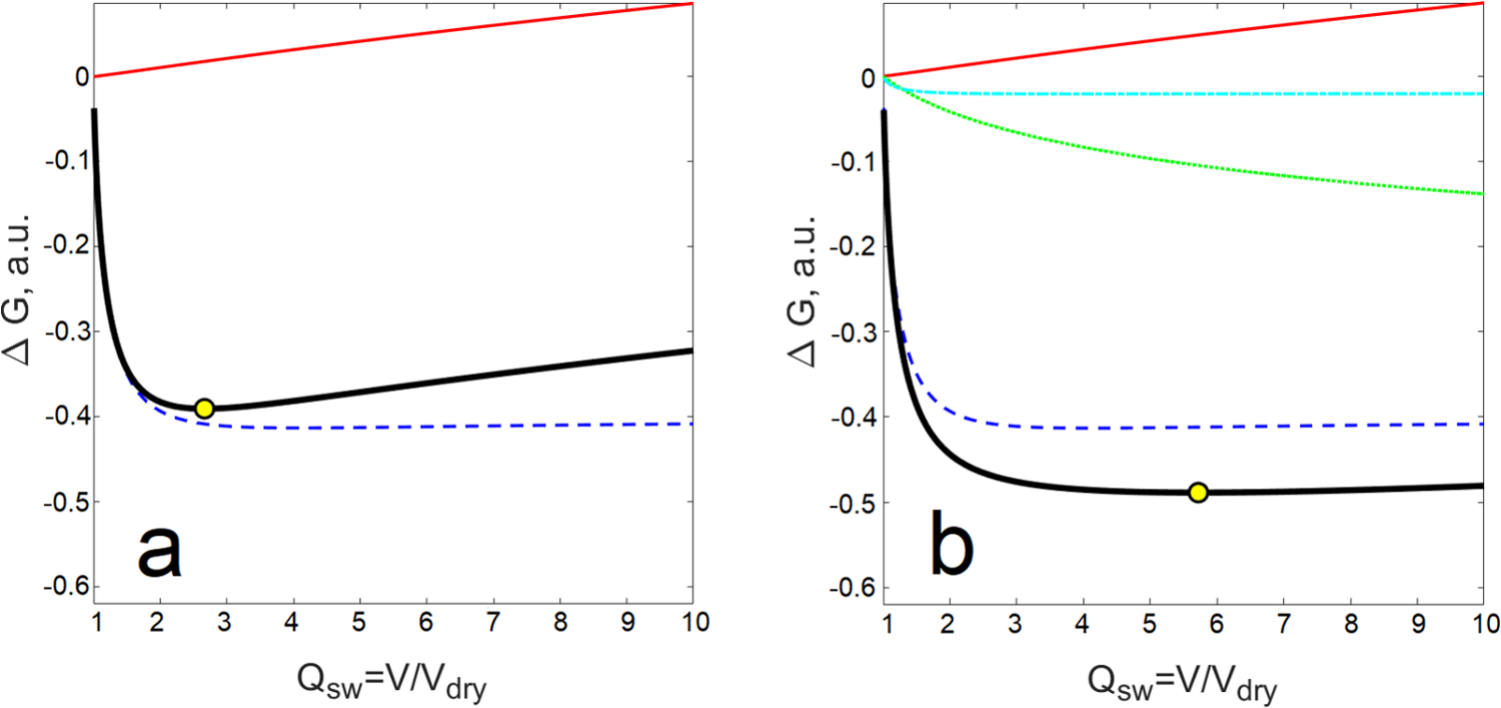
Schematic graph illustrating the swelling equilibrium (change in the Gibbs free energy of the hydrogel relative to its dry state, Δ*G*, as a function of the volume swelling ratio, *Q*_sw_ = *V*/*V*_dry_) of the neutral hydrogel in the absence (a) and presence (b) of ionic surfactant. In neutral hydrogels, the Gibbs free energy of the hydrogel (black line) is assumed to consist of mixing (blue dashed line) and an elastic term (red line). In the presence of ionic surfactant terms due to polymer–surfactant interactions (light blue dashed-dotted line), the effect of counterions dragged inside the hydrogel (green dotted line) must also be considered. The equilibrium swelling state corresponding to the minimum of Gibbs free energy for both cases is depicted as Ο.

The equilibrium volume and structure of the neutral polymer hydrogels are governed by the interaction of the polymer network building blocks with water and the elasticity of the network, respectively, as illustrated in Figure 1a. With increasing hydrogel volume, the network chains are stretched, increasing the Gibbs free energy. In contrast, this is accompanied by an increase of the number of thermodynamically favorable polymer–water contacts and a decrease of the Gibbs free energy. The swelling equilibrium corresponds to the minimum of the Gibbs free energy of the system when both the effects are mutually balanced.

POE and POP also differ in their interactions with ionic surfactants in aqueous solutions.^41−48^ This difference plays a significant role in the self-assembly of POE/POP block copolymers in aqueous surfactant solutions.^48−50^ The interaction between hydrophobic alkyl tails of the surfactant and polymer chains is very important in the formation of surfactant micelles around the polymer chains when the surfactant concentration exceeds a critical value (critical association concentration).^41^ Unlike POE, which is soluble in water at ambient temperature, POP is more soluble in hydrocarbons than in water. Therefore, POP exhibits a strong binding with the hydrophobic alkyl tails of ionic surfactants.^41,42^ Unlike this, POE is quite indifferent to these surfactants at ambient temperature;^41,51^ however, the interaction becomes more important at elevated temperatures with increasing hydrophobicity of the POE.^52^

When POP is built in the polymer network, swelling of the network in an aqueous solution of an ionic surfactant brings alkyl surfactant tails into the hydrogel to bind with POP. This binding further decreases the Gibbs energy of the hydrogel, as shown in Figure 1b. On the other hand, the surfactant tails are terminated by charged headgroups, and consequently, to conserve the electroneutrality of hydrogel, free highly movable counterions from dissociated surfactant molecules are also dragged inside the hydrogel. In this way, a neutral hydrogel is converted into an “ionic” hydrogel. If the surfactant tails self-organize in the hydrogel (similar to that in supercritical solutions), e.g., they form micelles, the charge density on the micelle surface becomes so high that some of the counterions condense onto the surface to reduce the net charge. The rest of the counterions remain highly mobile, and as they are confined in the limited volume of the hydrogel, they further expand the polymer network to increase the available space for their movement and significantly decrease the free energy of the system^21^ (see Figure 1b). Thus, swelling experiments are very sensitive to the presence of surfactants and represent a simple but sensitive method to study polymer–surfactant interactions.^25^ It was found that the surfactant effect on the swelling behavior of a polymer network is influenced by the presence of hydrophobic domains in the resulting hydrogel.^53^ In this case, the bound surfactant molecules penetrate the hydrophobic domains and alter their structure as demonstrated experimentally.

In our previous study,^35^ stoichiometric epoxy APN was prepared by the end-linking reaction of α,ω-diamino-terminated POP with an average molar mass of 2000 g·mol^–1^ and with POE bis(glycidyl ether) with an average molar mass of 526 g·mol^–1^. The POP and POE contents in the network were 66 and 34 wt %, respectively. The swelling behavior of the network in solutions of the cationic surfactant (myristyl triammonium bromide (C_14_TAB)), composition, and structure of the resulting hydrogels were investigated gravimetrically and by SANS. The nanophase-separated structure of the hydrogel prepared in the absence of a surfactant consisting of water-poor and water-rich domains was revealed. The characteristic length scale of the nanophase-separated structure, as measured by Bragg’s distance, is ca. 78 Å. This structure is preserved in the presence of the surfactant; however, it becomes finer because the magnitude of Bragg’s distance decreases from 78 Å (in the absence of the surfactant) to 61 Å (highest surfactant concentration).

In this study, a combination of gravimetry and the SANS was used. A new stoichiometric epoxy network was prepared using a longer POP (molar mass of ca. 4000 g·mol^–1^) and the same POE bis(glycidyl ether). Consequently, the cross-linking density of the present network is lower, and the POP and POE contents are 81 and 19 wt %, respectively. The effects of both the cationic (C_14_TAB) and anionic surfactants (sodium dodecyl sulfate (SDS)) on the composition and structure of the resulting hydrogels are investigated and compared. The importance of POP block length was evaluated. The results are expected to provide new information about the surfactant organization inside the polymer hydrogels, which is important for their practical applications.

## Materials and Methods

### Epoxy Networks

**Scheme 1:**
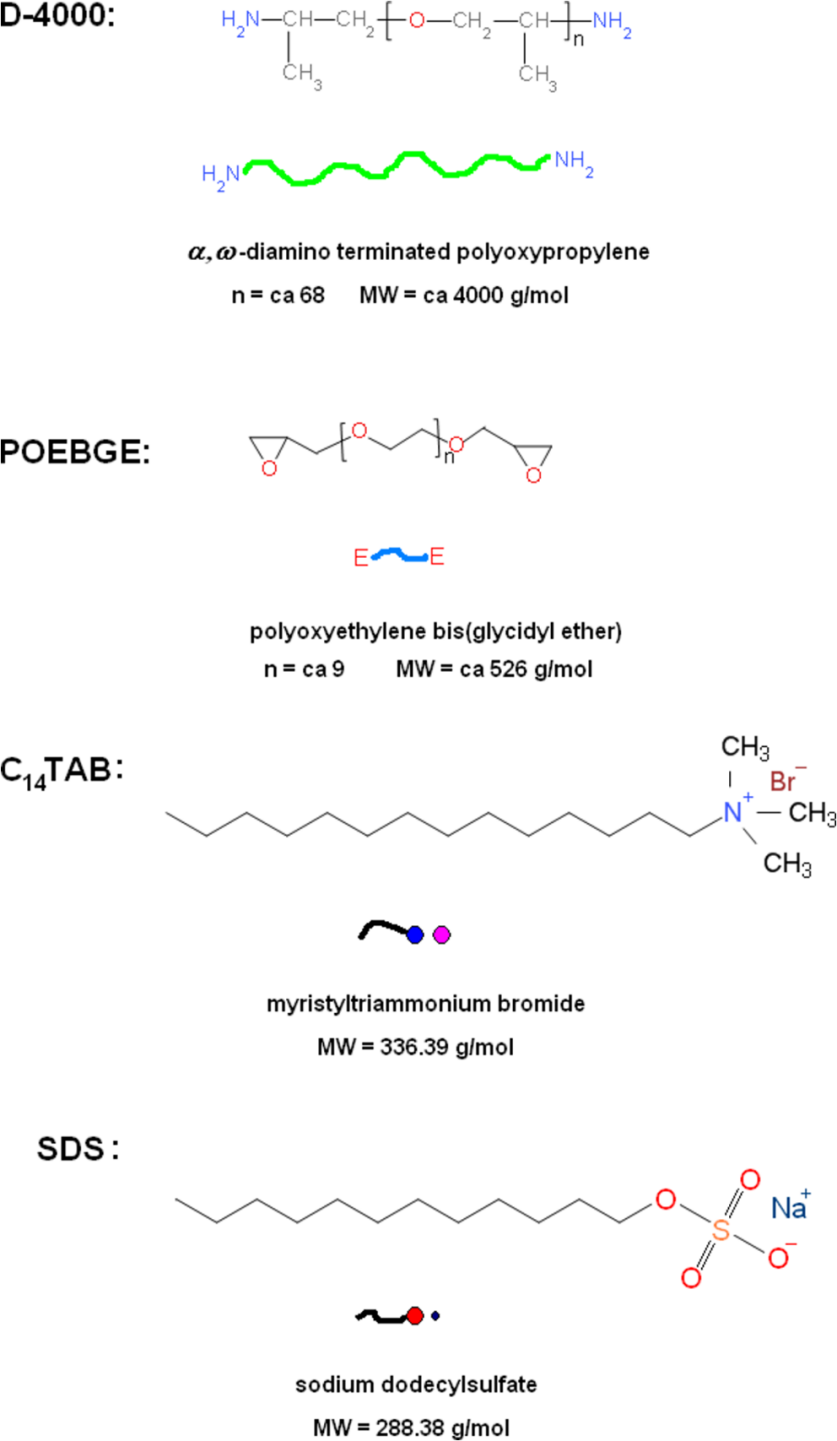
Chemical Structure and Representation of the Compounds Used in the Network and Hydrogel Preparation

The epoxy network used in this study was prepared by the end-linking reaction of α,ω-diamino-terminated POP (Jeffamine D-4000, Huntsman) with an average molar mass of 4000 g·mol^–1^ and POE bis(glycidyl ether) (POEBGE, Sigma-Aldrich) with an average molar mass of 526 g·mol^–1^. The chemical formulae of the reagents used are shown in Scheme 1. The network was stoichiometric, i.e., the initial molar ratio of amino and epoxy groups (stoichiometric ratio), *r* = 2[NH_2_]_0_/[E]_0_ = 1.00, where [NH_2_]_0_ and [E]_0_ are the initial molar concentrations of the amino and epoxy groups, respectively. The concentrations of amino and epoxy groups in the Jeffamine D-2000 and POEBGE determined by titrations were *c*_NH_2__ = 0.453 × 10^–3^ mol·g^–1^ and *c*_E_ = 3.86 × 10^–3^ mol·g^–1^, respectively. Both components were first stirred at 100 °C for about 15 min and then poured into Teflon molds. The curing reaction proceeded at 110 °C for 48 h. The network prepared was transparent. A small amount of extractable fraction sol (ca. 10 wt %) remaining in the network after the curing process was removed by triple extraction in a good solvent (toluene) at room temperature. Finally, the network was dried carefully, first in open air and then in a vacuum oven at 40 °C for 48 h.

### Swelling Behavior and Composition of Hydrogels

The swelling behavior and composition of the hydrogels were determined by the procedure used in our previous paper.^35^ Rectangular specimens with dimensions of ca. 10 × 10 × 1 mm^3^ were cut from the extracted and dry epoxy networks, and their masses were determined using a precise balance. The specimens were then swollen to equilibrium in a large excess of both subcritical and supercritical C_14_TAB and SDS solutions in D_2_O to obtain the hydrogels. The critical micelle concentrations (CMCs) of C_14_TAB and SDS in D_2_O at 25 °C are 3.2 and 7.6 mmol·L^–1^, respectively.^54^ The hydrogels prepared were termed C_14_TAB and SDS hydrogels, respectively. Swelling was carried out in a thermostated bath at 25 °C for the time required to attain equilibrium. The presence of long surfactant molecules such as C_14_TAB and SDS in the swelling solution significantly decelerates the swelling process. We have found experimentally that at room temperature approximately 3 weeks are necessary to attain swelling equilibrium of the specimens cut from the epoxy network in aqueous solutions of C_14_TAB and SDS (unlike ca. 2 days in neat water). The amount of swelling solution was sufficiently large to keep the amount of surfactant absorbed by the hydrogels negligible relative to the amount remaining in the swelling solution. Then, the hydrogels were removed from the solutions, quickly surface-dried, and their masses were determined. The specimens were then placed on Petri dishes and dried, first in open air and then in a vacuum oven at 40 °C for 48 h. The mass of the dried specimens was determined again.

Hydrogels consist of the polymer network, D_2_O, and absorbed surfactant, i.e., the mass of a hydrogel specimen, *m*_sw_, is given by
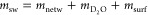
1where *m*_netw_, *m*_D_2_O_, and *m*_surf_ are the masses of the polymer network, D_2_O, and surfactant, respectively.

After drying, the hydrogel specimen consists of the polymer network and absorbed surfactant only, i.e., its mass, *m*_1_, is given by
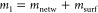
2

The mass fractions of the polymer network, *w*_netw_, surfactant, *w*_surf_, and water, *w*_D_2_O_, in hydrogels are then calculated using eqs 1 and 2
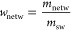
3
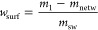
4
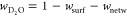
5

The swelling degree of the polymer network is given by
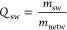
6

The surfactant uptake (in mass of the surfactant per mass of the dry network) is calculated as
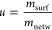
7

The molar surfactant concentration inside the hydrogel is calculated as
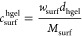
8where *d*_hgel_ is the specific mass of the hydrogel containing the surfactant and *M*_surf_ is the molar mass of the surfactant. Only small variations in the specific mass of the hydrogel with surfactant content are expected; for simplicity, in eq 8,*d*_hgel_ = 1.05 g·cm^–3^ is assumed in the calculations for all samples.

### Small-Angle Neutron Scattering

SANS measurements were carried out on a YuMO small-angle instrument in the IBR-2 pulsed reactor (JINR, Dubna, Russia) in the time-of-flight regime. A two-detector setup with ring wire detectors was used in the YuMO small-angle spectrometer.^55^ The neutron wavelength range was 0.05–0.8 nm. The hydrogels were extracted from the solutions immediately before measurements and placed in specific sample cells between two 1 mm round quartz discs. Cell construction was performed to exclude water outflow and evaporation during experiments. The temperature during the measurements was held constant at 25 °C by using a Lauda thermostat operating with the sample holder.

The measured scattering curves were corrected for the background scattering from an empty cell. For an absolute calibration of the scattering intensity during the measurements, a vanadium standard was used while silver behenate sample was used to calibrate distances.^56^ Treatment of the raw data was performed by the SAS program.^57^

### Fitting Procedure

The fitting of the SANS experimental curves was performed using Python (www.python.org) combined with the SciPy and NumPy packages (https://numpy.org). The procedure implied the consecutive use of the differential evolution global optimization method.^58^ Additional MCMC sampling with the emcee package^59^ was used to check the parameter correlations. For the calculation of the model curves, structure factors were introduced using the SASView (https://sasview.org) library. All the form factors, structure factors, and polydispersity approaches were tested in comparison with model computations in SASView.

## Results and Discussion

### Swelling Behavior and Composition of the Hydrogels

**Figure 2:**
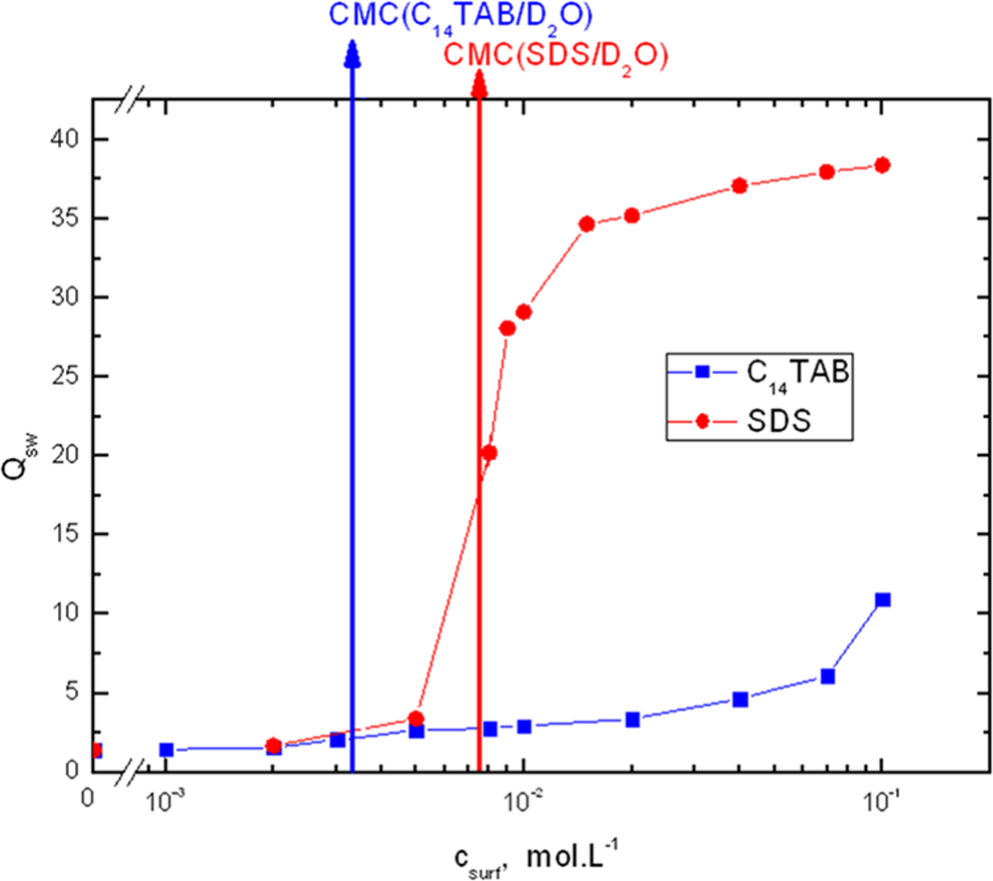
Dependence of equilibrium swelling ratio of the epoxy network on the surfactant concentration in swelling solution at 25 °C.

The dependence of the equilibrium swelling ratio of the network, *Q*_sw_, on the surfactant concentration in the surrounding swelling solution, *c*_surf_, for both surfactants is shown in Figure 2.

In the subcritical region, an almost equal growth of the swelling ratio with increasing surfactant concentration is observed for both surfactants. In the supercritical region, the growth continues for both surfactants; however, for SDS, the growth becomes very high in proximity to the critical micelle concentration, providing SDS hydrogels with much higher swelling degrees than C_14_TAB hydrogels. This dependence resembles the swelling behavior of ionic hydrogels, which is very sensitive to the concentration of counterions dragged by charges chemically firmly attached to the polymer network. Under proper conditions, a discrete transition from a less swollen (collapsed) to a highly swollen state is observed.^19^ As explained in the Introduction section, if the surfactant tails self-organize and form micelles, a part of the counterions condense on the micelle surface to reduce the net micelle charge. In supercritical aqueous solutions, the degree of micelle ionization, *α*, is higher for SDS (*α* = 0.35)^60^ than for C_14_TAB (*α* = 0.24),^60^ although there is some inconsistency in the *α* values reported by various authors.^61^ However, very high values of the swelling degree of the SDS hydrogels indicate that more counterions (sodium cations) remain mobile after being dragged inside the hydrogel than in the case of the C_14_TAB hydrogels (bromine anions). The difference in ion mobility can be attributed to the difference in the ionic radii of the sodium cations (1.29 Å) and bromine anions (1.95 Å)^62^ and differences in the size of their hydration shells.

**Figure 3:**
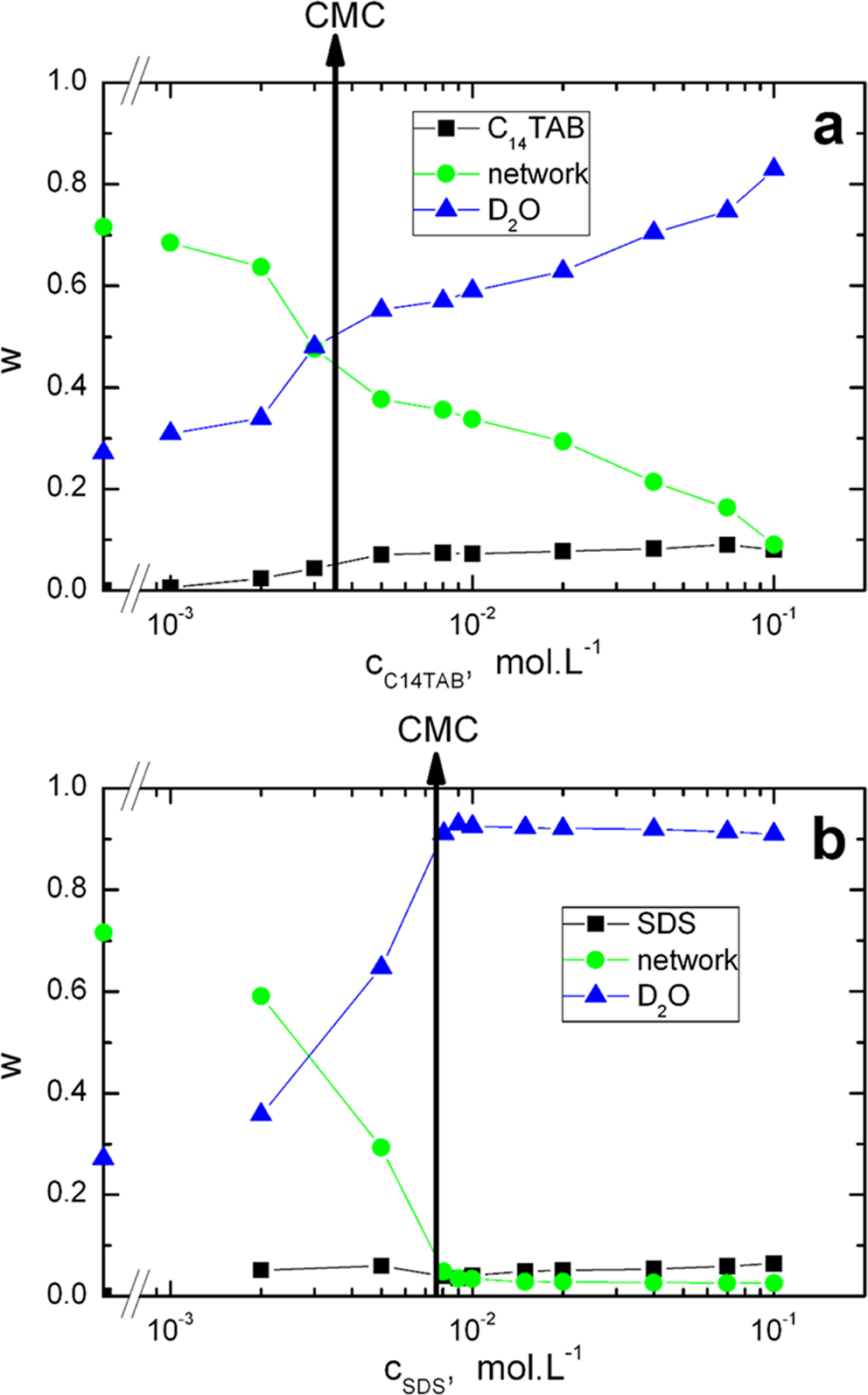
Dependence of the mass fractions of hydrogel components on the concentrations of C_14_TAB (a) and SDS (b) in the swelling solution at 25 °C.

Changes in the composition of the hydrogels (contents of the polymer network, D_2_O, and surfactant) with increasing surfactant concentrations in the swelling solution are shown in Figure 3a (C_14_TAB) and b (SDS), respectively.

For the C_14_TAB hydrogels, the polymer network content decreases from ca. 71 wt % (in neat D_2_O) to ca. 45 wt % at CMC, decreasing less in the supercritical region approaching ca. 9 wt % at the highest C_14_TAB concentration. For the SDS hydrogels, the decrease of the polymer network content is larger (ca. 4 wt % at CMC); in the supercritical region, it remains almost constant (ca. 3 wt %).

In the case of C_14_TAB hydrogels, the water content increases continuously from ca. 29 wt % (in neat D_2_O) to ca. 50 wt % at CMC; in the supercritical region, the growth becomes less pronounced approaching ca. 82 wt % at the highest C_14_TAB concentration (0.1 mol·L^–1^). In contrast, for the SDS hydrogels, the water content increases significantly, approaching ca. 90 wt % at CMC; in the supercritical region, it becomes almost constant (91–93 wt %).

For the C_14_TAB hydrogels, the surfactant content increases continuously to ca. 8 wt % at CMC, remaining almost constant at this value in the supercritical region. For the SDS hydrogels, an increase of ca. 6 wt % is observed in the subcritical region, followed by a drop to ca. 4 wt % at the CMC (caused by the high expansion of the hydrogel) and a slow increase of ca. 6 wt % again in the supercritical region.

**Figure 4:**
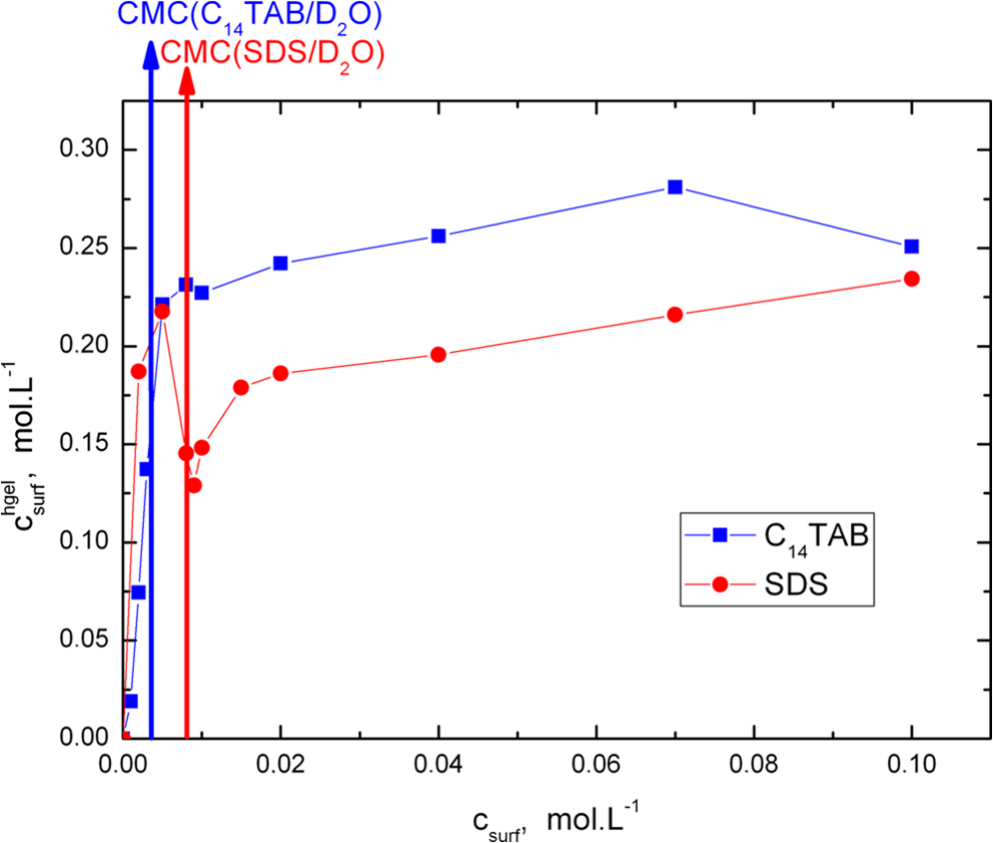
Dependence of the surfactant concentration in hydrogels on the surfactant concentration in the swelling solution at 25 °C.

Figure 4 shows a comparison of the surfactant concentrations in the hydrogel and swelling solution used in its preparation. For both surfactants, the concentrations determined in the hydrogels are much higher than those determined in the surrounding swelling solutions. This is in agreement with the thermodynamically favorable mixing of the hydrophobic alkyl surfactant tails with the POP chains in the polymer network. In the subcritical region, both the C_14_TAB and SDS concentrations in the hydrogel increase with increasing surfactant concentration in the swelling solution and obtain comparable values. In the supercritical region, the C_14_TAB concentration in the hydrogel increases further, but more slowly than in the case of SDS. The lower final value at the highest C_14_TAB concentration than expected may be attributed to a change in the hydrogel structure at the highest C_14_TAB concentration. For SDS at the CMC, a drop in its concentration in the hydrogel is observed, which can be attributed to the huge expansion of the hydrogel volume observed. In the supercritical region, the SDS concentration increases further; however, it is less pronounced than that in the subcritical region of concentrations.

**Figure 5:**
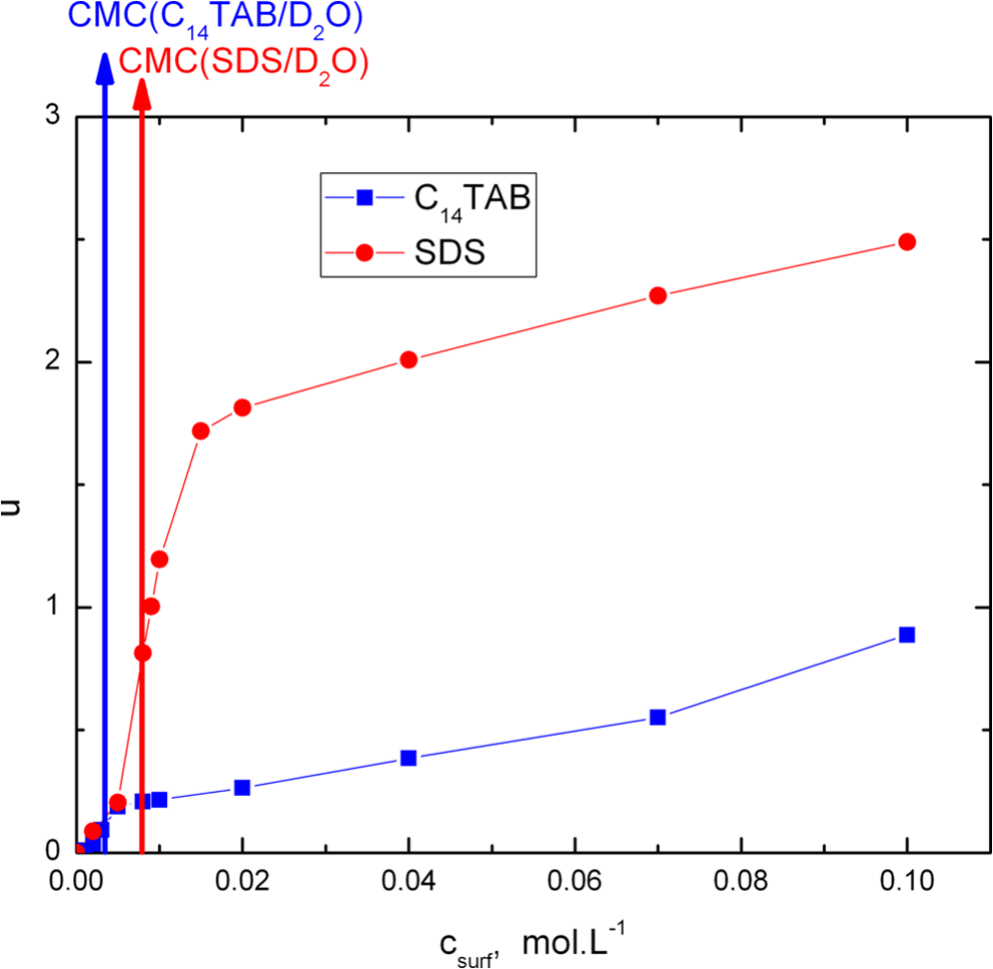
Dependence of surfactant uptake (surfactant/polymer network mass ratio) on the surfactant concentration in swelling solution at 25 °C.

Finally, Figure 5 shows the uptakes of the two surfactants by the polymer network. In the subcritical region, the uptake of both surfactants increases with the surfactant concentration in the swelling solution and obtains almost equal values. In the supercritical region, the increase of the uptake becomes less pronounced for both surfactants; however, for SDS, it is much larger than that for C_14_TAB.

### SANS Profiles

**Figure 6:**
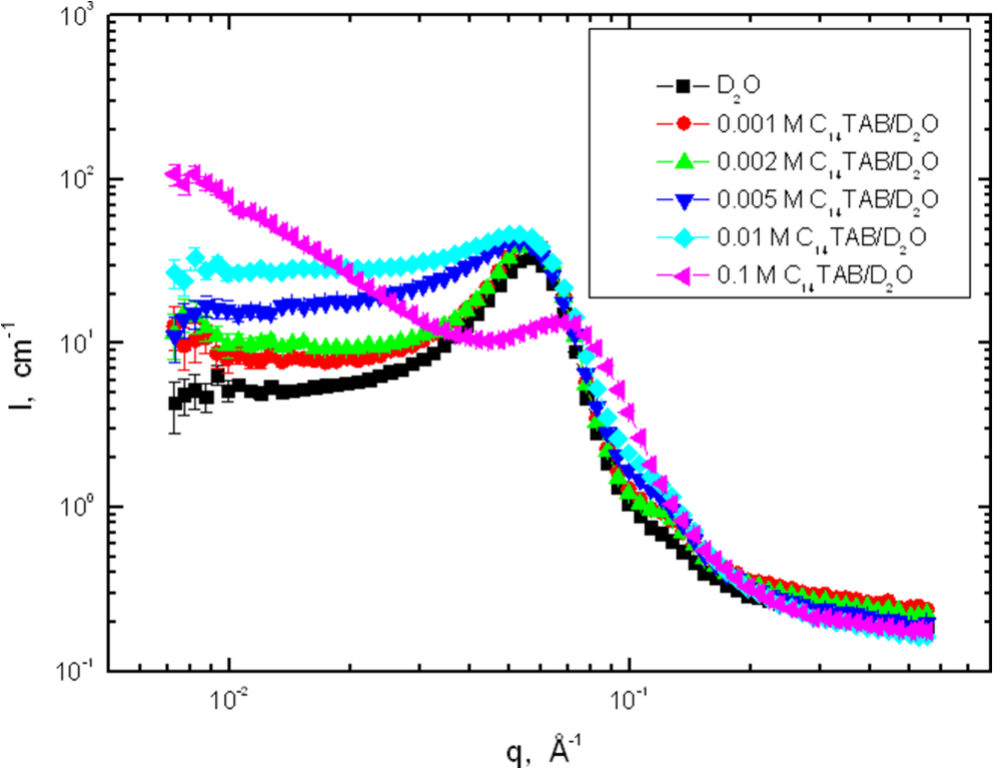
Log–log plot of SANS profiles (scattering intensity *I* vs magnitude of the scattering vector *q*) obtained from the epoxy network swollen to equilibrium in C_14_TAB/D_2_O solutions at 25 °C.

Figure 6 shows the SANS profiles collected from the hydrogels obtained by swelling the epoxy network in D_2_O and C_14_TAB solutions in D_2_O.

The features shown in Figure 6 can be summarized as follows: (1) a distinct scattering peak is observed for all hydrogels. (2) The position of the peak moves only slightly from ca. 0.057 Å^–1^ (in D_2_O) to 0.053 Å^–1^ (in 0.01 mol·L^–1^ C_14_TAB). For the highest surfactant concentration (0.1 mol·L^–1^ C_14_TAB), the peak is lower and of a changed shape, and its position is shifted to 0.068 Å^–1^ and the low-*q* intensity becomes stronger. (3) The scattering peak becomes broader at the low-*q* side with increasing surfactant concentration. (4) Except for the highest surfactant concentration, the scattering knee located at ca. 0.12 Å^–1^ is also visible and its intensity is magnified by the presence of the surfactant.

**Figure 7:**
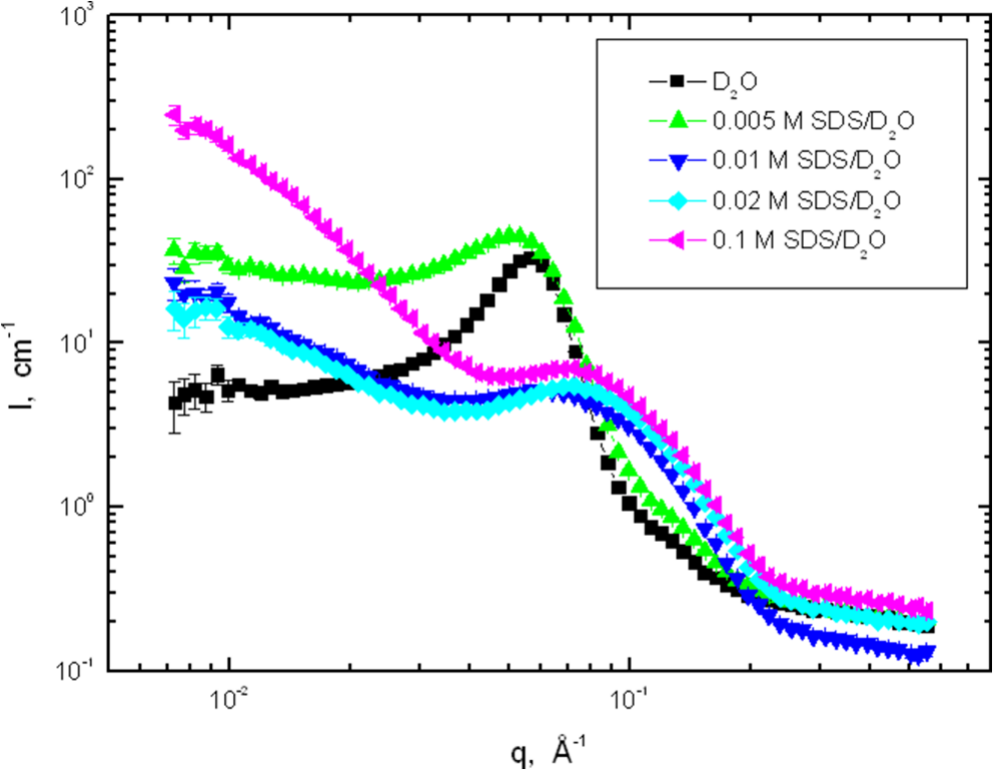
Log–log plot of SANS profiles obtained from the epoxy network swollen to equilibrium in SDS/D_2_O solutions at 25 °C.

Figure 7 shows the SANS profiles collected from the hydrogels obtained by swelling the epoxy network in D_2_O and SDS solutions in D_2_O.

The features shown in Figure 7 can be summarized as follows: (1) a distinct scattering peak is observed again for all hydrogels. (2) In the subcritical region of the surfactant concentration, the position of the peak shifts from ca. 0.057 Å^–1^ (in D_2_O) to 0.050 Å^–1^ (in 0.005 mol·L^–1^ SDS), and it becomes much wider on the low-*q* side with increasing surfactant concentration. (3) In the supercritical region of surfactant concentrations, the peak position is shifted to the higher-*q* region (0.060 and 0.073 Å^–1^ for 0.01 and 0.1 mol·L^–1^ SDS, respectively). The peak is much broader than that in the subcritical region, and the low-*q* intensity becomes stronger. The shape of the peak resembles that observed for micelles formed in aqueous solutions of ionic surfactants; see e.g., refs (63) and (64). (4) In the subcritical region, the scattering knee located at ca. 0.12 Å^–1^ is visible, and its intensity is magnified by the presence of the surfactant.

Figures S3 and S4 show coherent SANS profiles obtained by the subtraction of a constant background (including incoherent) scattering, *I*_B_, determined by fitting of the SANS profile tails (for 0.2 Å^–1^ < *q* < 0.4 Å^–1^) using eq 9 and assuming
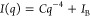
9where *C* is Porod’s constant. Figure S5 shows Porod fits in this region. Since the scattering at *q* > 0.4 Å^–1^ starts to reflect structural details shorter than a few Ås that are not of interest in this study, they were excluded from the fitting in this region. They are assumed to contribute to the background scattering.

The positions of the scattering peak, *q*_max_, presented in Figures 6 and 7, and the corresponding values of Bragg’s distance *D*_B_, defined by *D*_B_ = 2π/*q*_max_, are summarized in Table 1. From these values, it can be concluded that the hydrogels exhibit variations in the neutron scattering length density on the characteristic scale of 86–126 Å. The value of Bragg’s distance determined for the hydrogel in the absence of a surfactant is 110 Å. This is approximately √2 times the *D*_B_ value determined in the hydrogel obtained by swelling the epoxy network containing 2 times shorter POP (*M*_n_ = 2000 g·mol^–1^),^35^*D*_B_ = 78 Å. This value can also be compared with the value of the root of the mean-squared end-to-end distance of the POP chain. For a molar mass 4000 g·mol^–1^, one obtains ⟨*r*^2^⟩^1/2^ ≈ 46 Å (see Section S2 in the Supporting Information). Thus, *D*_B_/⟨*r*^2^⟩^1/2^ ≈ 2.36.

**Table 1:** Composition of Hydrogels and Parameters Determined by a General Analysis of SANS Profiles^a^

*c*_surf_ (mol·L^–1^)	*v*_D_2_O_	*v*_POE_	*v*_POP_	*v*_surf_	*q*_max_ (Å^–1^)	*D*_B_ (Å)	*l*_P_ (Å)	*Q*_INV_ (10^20^ cm^–4^)
D_2_O								
0	0.271	0.131	0.598	0	0.057	110	40	39
C_14_TAB								
Subcritical								
0.001	0.293	0.126	0.573	0.008	0.057	110	40	49
0.002	0.320	0.116	0.531	0.033	0.057	110	39	49
Supercritical								
0.005	0.521	0.069	0.313	0.097	0.057	110	40	56
0.01	0.557	0.062	0.281	0.100	0.053	119	39	69
0.1	0.794	0.017	0.076	0.113	0.068	92	28	49
SDS								
Subcritical								
0.005	0.617	0.054	0.246	0.083	0.050	126	42	61
Supercritical								
0.01	0.906	0.007	0.029	0.058	0.057	110	20	35
0.02	0.897	0.005	0.024	0.074	0.073	86	18	45
0.1	0.882	0.005	0.022	0.091	0.073	86	17	58

a *v*_D_2_O_, *v*_POE_, *v*_POP_, and *v*_surf_: volume fractions of D2O, POE, POP, and surfactant, and parameters determined by a general analysis of SANS profiles: *q*_max_, the position of the scattering maximum; *D*_B_, Debye’s length; *l*_P_, Porod’s length; and *Q*_INV_, scattering invariant..

### Scattering Invariant

To obtain more information on the nanophase-separated structure of the hydrogels, values of the scattering invariant, *Q*_INV_, were calculated by integration of the coherent part of the scattering intensity over the entire *q*-range as
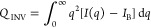
10where *I*_B_ is the background scattering determined from Porod’s fit (see eq 9). The *Q*_INV_ values of the hydrogels are listed in Table 1.

### Porod’s Length of Inhomogeneity

Except for the hydrogels with broad scattering maxima, the values of Porod’s length of inhomogeneity, *l*_P_, were calculated as
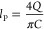
11

Porod’s length^65^ is a measure of the average size of the phases. For phases separated by sharp boundaries, it is defined as a harmonic mean of the average chord length of individual phases I and II by
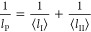
12

Of course, Porod’s length is smaller than any of the average chord length:
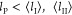


The calculated values of this parameter are listed in Table 1.

For hydrogels with a narrow scattering peak, Porod’s length obtained similar values between 39 and 42 Å, which represent about 0.35–0.36 *D*_B_-values. Similar to the *D*_B_-values, the *l*_P_-values determined for the present C_14_TAB hydrogels are approximately √2 times the *l*_P_-values determined in the previous system where twice shorter POP was used (*l*_P_ between 20 and 28 Å).^35^ Smaller magnitude of Porod’s length determined for the hydrogels with a broad peak (from 17 to 28 Å) indicates that the two-phase structure becomes finer. The formation of similar broad peaks was not observed in the systems studied previously.^35^

### Fitting of SANS Profiles

The experimental SANS profiles presented in Figures 6 and 7 show the two-phase structure of the hydrogels. To perform fitting of SANS profiles mathematical expressions for scattering intensity derived from a more detailed information structure and the dynamics of the systems are needed.

In hydrogels showing a narrow scattering peak, a bicontinuous morphology of the phases is possible. Teubner–Strey model^66^ is the only available model providing mathematical expression for small-angle scattering for this kind of morphology. However, the Teubner–Strey model provides a scattering peak that is too wide and fails to fit the SANS profiles from the hydrogels presented in this study. Another option is to approximate the hydrogel morphology by a system consisting of (polydisperse) spherical particles of one phase interacting via a hard-sphere (HS) potential in the Percus–Yevick (PY) approximation and being dispersed in a matrix of the other phase. The effective radius of the HS interaction between particles is assumed to be larger than or equal to the particle radius. This model is often used in the analysis of SAXS/SANS profiles from various micro- and nanophase-separated systems; see, e.g., ref (67). It was also used successfully in the fitting of SANS from epoxy-based hydrogels in one of our previous study.^30^

With regard to the hydrogels showing a broad scattering peak, conclusions drawn from the shape of the scattering peak support the idea that the hydrogel structure consists of the phase of micelles of aggregated surfactant molecules dispersed in the swollen polymer network, representing the other phase. Then, mathematical expressions derived for scattering profiles from micelles of ionic surfactants with mutual interactions described by the rescaled mean spherical approximation (RMSA) can be used.

Thus, the expression for the scattering intensity for both cases can be expressed as
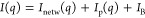
13where *I*_netw_(*q*), *I*_p_(*q*), and *I*_B_ are the contributions from the swollen network, particles of one of the phases (spherical domains or charged micelles) dispersed in a matrix, and the background scattering, respectively.

For the contribution from the swollen polymer network, we will use the Debye–Bueche (Debye–Anderson–Brumberger) formula in the form
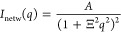
14where *A* is a constant and Ξ is the correlation length.

The contribution from a system of polydisperse spherical particles will be expressed by^68^
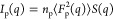
15where *n*_p_ is the number concentration of the particles, *F*_p_(*q*) is the particle scattering form factor, and *S*(*q*) is the structure factor. The symbol <···> denotes the average of the distribution of sphere sizes. For polydisperse systems, the number concentration of the spherical particles, *n*_p_, is related to their volume fraction, *v*_p_, by
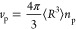
16

The scattering form factor for spherical particles of radius *R* will be used (for definition see ref (68)).
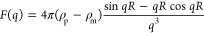
17where *ρ*_p_ and *ρ*_m_ are the scattering densities of the particle and matrix phases, respectively.

A log-normal distribution function (see, e.g., ref (69)), *p*(*R*), will be used for the description of sphere radii distribution
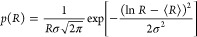
18where ⟨*R*⟩ is the mean radius of spheres, and *σ* is the root-mean-square deviation (polydispersity) of the sphere radii.

The two fitting models differ in their expression of the structure factor. The structure factor for the HS interaction in the PY approximation was used for the interaction between spherical domains and the rescaled mean spherical approximation (RMSA)^70^ was used for the interaction between electrically charged spherical particles. A single effective interaction radius, *R*_eff_, greater than or equal to the radius of the spheres, is assumed in the models. For the mathematical formulae for *S*(*q*) for the HS and RMSA models, we refer readers to refs (67) and (70).

When the RMSA structure factor was used for the hydrogels swollen in supercritical SDS solutions, a strong correlation was observed between the values of the scattering contrast, |Δ*ρ*| ≡ |*ρ*_p_ – *ρ*_m_|, and the volume fraction of particles. This was further analyzed by separately fitting the experimental SANS curves using different fixed values of |Δ*ρ*|. In a wide range of |Δ*ρ*|-values, the fit quality in terms of *χ*^2^, as well as visual agreement, was very good. Thus, the corresponding fitting parameters obtained are presented as ranges of acceptable values (Table 2).

**Table 2:** Best Parameter Values of HS and RMSA Models Determined by Fitting of SANS Profiles^a^

HS model						
sample	|Δ*ρ*| (10^10^ cm^–2^)	⟨*R*⟩ (Å)	*σ*	*R*_eff_ (Å)	*v*_p_	
D_2_O	4.57	38.9	0.154	54.8	0.128	
C_14_TAB						
Subcritical						
0.001 M	3.96	41.8	0.145	53.4	0.208	
0.002 M	3.88	42.2	0.137	53.5	0.212	
Supercritical						
0.005 M	4.14	44.8	0.163	50.2	0.257	
0.01 M	4.10	44.8	0.165	47.5	0.305	
SDS						
Subcritical						
0.005 M	3.66	41.2	0.149	53.4	0.196	

RMSA model						
sample	|Δ*ρ*| (10^10^ cm^–2^)	⟨*R*⟩ (Å)	*σ*	*R*_eff_ (Å)	*v*_p_	*q*_s_ (*e*)
C_14_TAB						
Supercritical						
0.1 M	3.71	27.7	0.230	32.0	0.25	2.3
SDS						
Supercritical						
0.01 M	6.36–6.06	16.7	0.243–0.248	16.7	0.055–0.061	7.3–7.1
0.02 M	6.36–6.16	16.3	0.250–0.253	16.3	0.081–0.087	6.5–6.4
0.1 M	6.36–6.06	16.5	0.271–0.277	16.5	0.109–0.122	11.8–12.1

a |Δ*ρ*| ≡ |*ρ*_p_ – *ρ*_m_|, the absolute value of the difference in the scattering length density between the particles and matrix (scattering contrast); ⟨*R*⟩, the average radius of particles; *σ*, polydispersity of radius of the particles; *R*_eff_, effective interaction radius; *v*_p_, the volume fraction of particles; and *q*_s_, the average charge per sphere (in the RMSA model).

**Figure 8:**
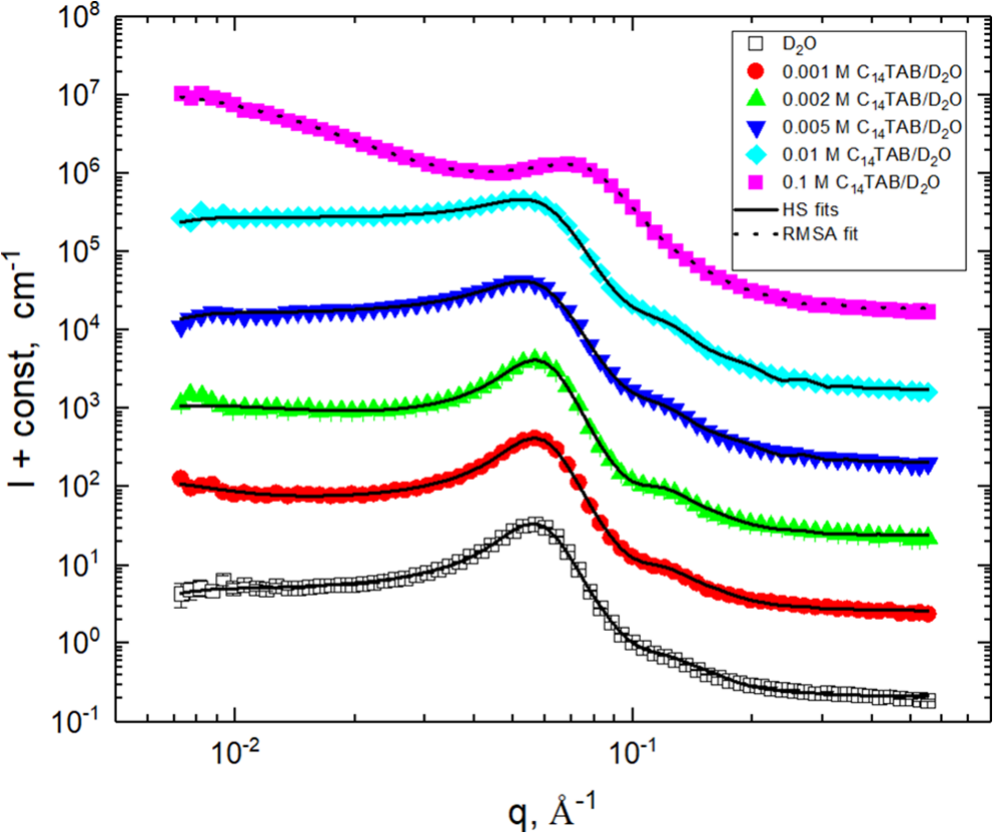
Comparison of experimental SANS profiles and best-fit curves for C_14_TAB hydrogels. Profiles are shifted for clarity.

**Figure 9:**
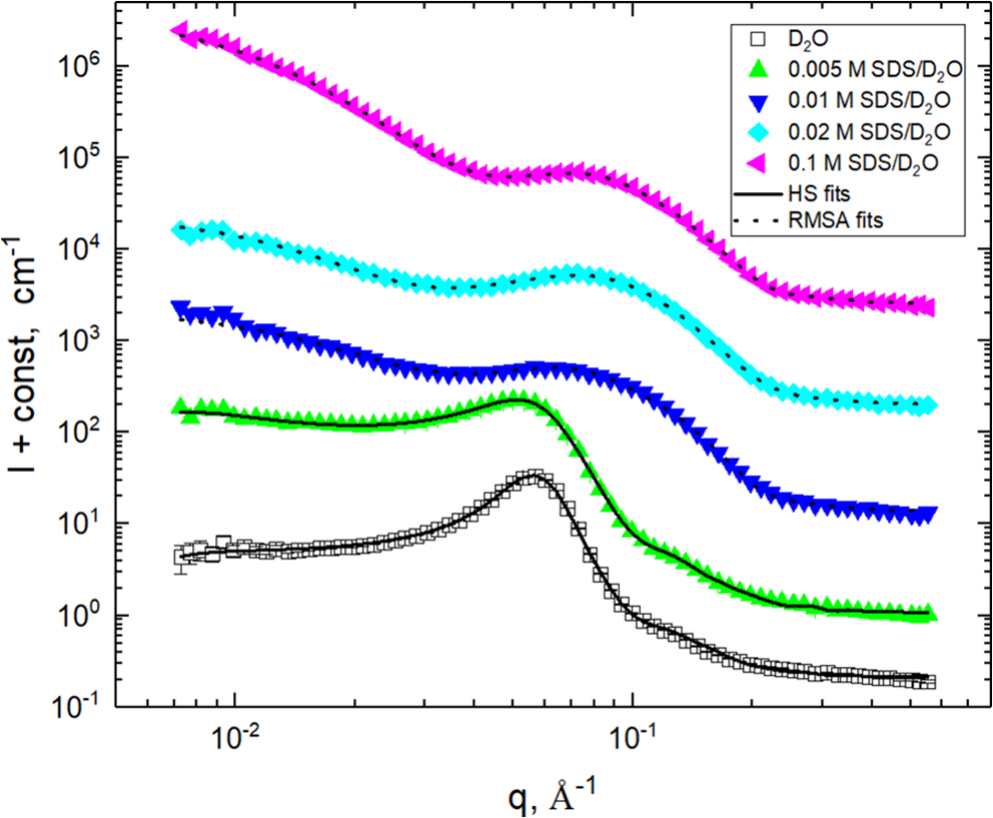
Comparison of experimental SANS profiles and best-fit curves for SDS hydrogels. Profiles are shifted for clarity.

The best-fit curves are shown in Figures 8 and 9 and the best-fit values of the HS and RMSA model parameters are summarized in Table 2.

### Nanoscale Structures of Hydrogels Consistent with SANS Results

The lowest-*q* scattering contribution is mainly due to static frozen inhomogeneities in the epoxy network formed during its preparation.^40^ This contribution is magnified by the swelling of the network; therefore, it increases with the surfactant concentration. However, as it does not contain relevant information about the structure of hydrogels at the nanoscale, it will not be discussed.

In the identification of the compositions of hydrogel structures consistent with the SANS data, the values of the scattering invariant, *Q*_INV_, determined by a general analysis of SANS data (see Table 1), and the absolute values of the difference of the scattering length density between particles and matrix, |Δ*ρ*| ≡ |*ρ*_p_*–ρ*_m_|, and volume fractions of particles (spherical domains or micelles), *v*_p_, obtained by fitting of SANS curves (see Table 2) will be used. For this purpose, the volume fractions of the hydrogel constituents, D_2_O, POE, POP, and surfactant (*v*_D_2_O_, *v*_POE_, *v*_POP_, and *v*_surf_) are also needed, and their values are calculated from the compositions of hydrogels (see Figure 3a,b), assuming additivity of volumes, are listed in Table 1. The scattering length densities of D_2_O, POE, POP, C_14_TAB, and SDS are listed in Table 3.

**Table 3:** Values of Parameters for SANS Evaluation^a^

compound	*d* (g·cm^–3^)	*ρ* (10^10^ cm^–2^)
D_2_O	1.10	6.35
POE	1.08	0.61
POP	1.01	0.35
alkyl tail	0.75^b^	–0.37
TA^+^ (cationic head of C_14_TAB)	0.67^c^	–0.30
Br ^–^ (anionic part of C_14_TAB)	3.10^d^	1.59
DS^–^ (anionic head of SDS)	1.83^e^	2.99
Na^+^ (cationic part of SDS)	0.93^f^	0.88

a Specific mass, *d*, and neutron scattering length density, *ρ*, of hydrogel components at 25 °C.

b Estimated using the specific masses of dodecane and tetradecane at 20 °C and 0.1 MPa.

c Estimated using the specific mass of liquid trimethylammonium at 0 °C and 0.1 MPa.

d Estimated using the specific mass of liquid bromine at 20 °C and 0.1 MPa.

e Estimated using the specific mass of liquid sulfuric acid at 20 °C and 0.1 MPa.

f Estimated using the specific mass of liquid sodium at 98 °C and 0.1 MPa.

For the hydrogel prepared by swelling in neat D_2_O, the volume fraction of particles determined by fitting using the HS model is *v*_p_ = 0.128. The largest part of this hydrogel consists of hydrophobic POP (*v*_POP_ = 0.598). Hydrophilic POE (*v*_POE_ = 0.131) and D_2_O (*v*_D_2_O_ = 0.271) form the rest of the hydrogel. Therefore, as the simplest model of the two-phase structure of this hydrogel, a particulate (minor) phase formed by POE mixed with D_2_O and a matrix (mayor) phase formed by POP are considered. However, this model provides a too high volume fraction of the particulate phase (*v*_POE_ + *v*_D_2_O_ = 0.402) and must be rejected. During network preparation, POE and POP chains are firmly covalently linked to the epoxy network. When immersed in water, the POE chains prefer to be in proximity to water molecules and, together with neighboring POP chains connected to them, might form a matrix phase. The remaining water (with a small amount of POE and POP) formed the particulate phase. To test this idea, we calculated the dependence of the volume fraction of the particulate phase, i.e., *v*_p_, the absolute value of the difference in the scattering length density between the phases, |Δ*ρ*|, and the scattering invariant as functions of the water content in one of the phases.

For this purpose, the volume fractions and scattering length densities of the particulate (p) and matrix (m) phases are calculated assuming volume additivity as
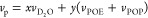
19
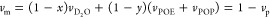
20and
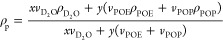
21
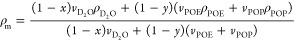
22where 0 ≤ *x* ≤ 1 and 0 ≤ *y* ≤ 1 are the fractions of D_2_O and epoxy network in the particulate phase, respectively. The scattering invariant (as defined in eq 9) of the idealized two-phase hydrogel with sharp interface boundaries is given by
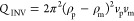
23

**Figure 10:**
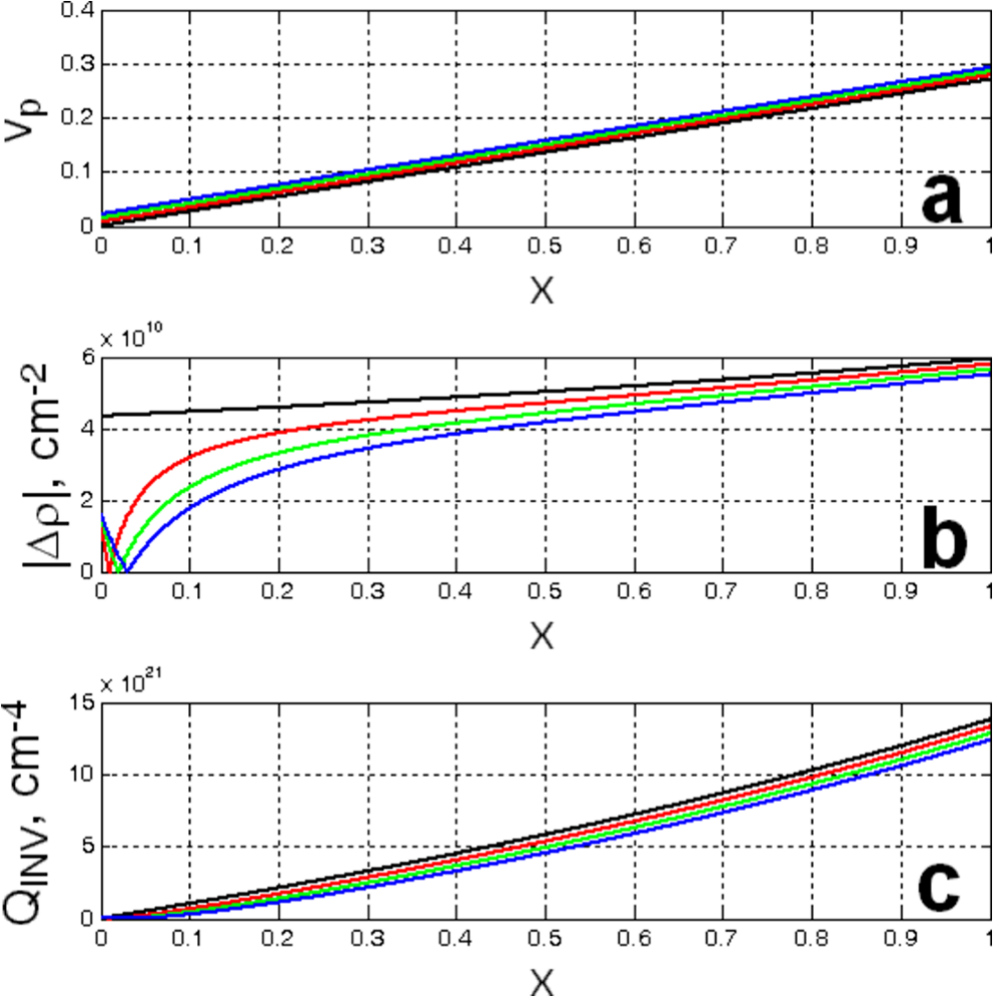
Dependences calculated assuming the two-phase structure of the epoxy network swollen in D_2_O, with phase I: *v*_I_ = *v*_p_ = *xv*_D_2_O_ + *y*(*v*_POE_ + *v*_POP_) and phase II: *v*_II_ = *v*_m_ = (1 – *x*)*v*_D_2_O_ + (1 – *y*)(*v*_POE_ + *v*_POP_). (a) *v*_p_ as a function of *x*, (b) |Δ*ρ*| as a function of *x*, and (c) *Q*_INV_ as a function of *x*, for a series of *y*-values: (black line) *y* = 0, (red line) *y* = 0.01, (green line) *y* = 0.02, and (blue line) *y* = 0.03.

If the particulate phase consisted of D_2_O only (i.e., *y* = 0), *v*_p_ = 0.128 obtained by fitting gives *x* = 0.47, i.e., about 47% of all D_2_O would be in this phase (see Figure 10a). However, in this case, the predicted value of the absolute scattering length density difference, |Δ*ρ*| = 4.97 × 10^10^ cm^–2^ (see Figure 10b), is too high compared to the value determined by fitting (|Δ*ρ*| = 4.57 × 10^10^ cm^–2^; see Table 2). To achieve better agreement, it is necessary to assume that a small amount of epoxy network must also be present in the particulate phase. Indeed, the best agreement is obtained when *y* ≈ 0.01, *x* = 0.44, and |Δ*ρ*| = 4.57 × 10^10^ cm^–2^ (see Figure 10b). Although, in this case, the predicted value of the scattering invariant *Q*_INV_ = 45.6 × 10^20^ cm^–4^ (see Figure 10c) is higher than that obtained by a general analysis of SANS data (*Q*_INV_ = 39 × 10^20^ cm^–4^; see Table 1), the difference is reasonable and can be explained by the nonsharpness of interface boundaries. The scattering invariant for a two-phase material with diffuse interface boundaries of effective thickness *t* is given by^65^
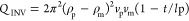
24

Since the magnitude of Porod’s length is small (*l*_P_ = 40 Å, see Table 1), the effective thickness of the magnitude, *t* = 5.8 Å, will give *Q*_INV_ = 39 × 10^20^ cm^–4^. To conclude, the structure of the hydrogel obtained by swelling the epoxy network in neat D_2_O, which is consistent with the SANS data, is represented by two phases divided by diffusive interface boundaries, both composed of D_2_O and the epoxy network; however, they differ strongly in the network content. In other words, the hydrogel consists of a highly swollen (particulate phase) and a poorly swollen epoxy network (matrix phase). The particulate phase contains about 44 vol % of all D_2_O and 1 vol % of all epoxy networks.

The domains of the particulate phase are well represented by polydisperse spheres with average radius ⟨*R*⟩ = 38.9 Å, and a low degree of polydispersity, *σ* = 0.154. The magnitude of the effective HS interaction radius, *R*_eff_ = 54.8 Å, is about 40% larger than the average sphere radius. This difference can be attributed to the presence of the polymer network chains in the matrix phase, hindering the spheres from approaching each other closer (to a distance equal to their diameter).

The presence of a subcritical amount of C_14_TAB in the swelling solution does not strongly change the position of the scattering peak and its shape on the high-*q* side in the C_14_TAB hydrogels (see Figure 6). Therefore, the two-phase structure of these hydrogels must be very similar to that of the hydrogel prepared in neat D_2_O; it should consist of the spherical domains of a highly swollen epoxy network dispersed in the matrix of the poorly swollen epoxy network. However, the phases also contain dissociated C_14_TAB molecules. Due to the excellent solubility of POP in hydrocarbons and its low solubility in water at 25 °C, the alkyl C_14_TAB tails are expected to be mixed dominantly with POP in the matrix phase, where the POP concentration is very high, and polar surfactant heads are located at the interface boundaries. Bromine counteranions are expected to be present in both phases, expanding them and increasing the D_2_O content in both phases. Considering the magnitudes of the scattering length densities of the dissociated parts of C_14_TAB (see Table 3), the presence of the surfactant should decrease |Δ*ρ*| and increase *v*_p_ and *Q*_INV_. This is confirmed experimentally (Tables 1 and 2). The structure is preserved even in hydrogels prepared in supercritical C_14_TAB solutions with concentrations lower than 0.1 M. The average radius of the spherical domains increases from 38.9 Å (in neat D_2_O) with increasing C_14_TAB concentration up to 44.8 Å (in 0.01 M C_14_TAB) and confirms this idea. The effective radius of the HS interaction, *R*_eff_, decreases slightly due to the higher swelling and, consequently, “softening” of the matrix phase.

Finally, at the highest C_14_TAB concentration, the shape of the scattering peak changed significantly and a new two-phase structure was formed. As the shape of the peak resembles that of the peaks registered in the C_14_TAB supercritical micelle solutions, this structure is expected to consist of similar micelles dispersed in the highly swollen epoxy network (matrix phase) containing the remaining dissociated C_14_TAB molecules and bromine counteranions. It is known that C_14_TAB (as well as SDS) micelles formed in the aqueous solutions are of rotational ellipsoidal form.^63,71^ For simplicity, to keep the number of fitting parameters as small as possible, the micelles in this paper are assumed to be polydisperse spheres of average radius ⟨*R*⟩. The values of the average radius ⟨*R*⟩ determined by fitting are compared with the values of the average cross-sectional radius of the ellipsoidal micelles reported in the literature.

The average radius of the micelles determined by fitting is 27.7 Å and is higher than the cross-sectional radius of ellipsoidal micelles found in supercritical C_14_TAB solutions (ca. 17 Å).^71^ The value of *v*_p_ ≈ 0.25 determined for this hydrogel exceeds the volume fraction of C_14_TAB in the hydrogel determined gravimetrically (*v*_surf_ = 0.113; see Table 1). Therefore, in addition to alkyl tails and polar headgroups, the micelles contain some additional material, most probably part of the POP chains from the swollen epoxy network in which they are dispersed. This idea is supported by the strong binding of POP to the alkyl tails of ionic surfactants observed in their mixed solutions.^51^ The average charge on the micelle surface is ca. 2.3 elemental charges. This value is much smaller than that determined for the C_14_TAB micelles in supercritical solutions (*q*_s_ ≈ 15 elemental charges).^71^

Similar to C_14_TAB, the presence of a subcritical amount of SDS in the swelling solution does not significantly change the position of the scattering peak and its shape on the high-*q* side (see Figure 7). Therefore, the two-phase structures of the subcritical SDS hydrogels and C_14_TAB hydrogels are very similar: alkyl tails mixed with the POP chains in the poorly swollen matrix phase and polar surfactant heads located at the interface boundaries. Sodium countercations are also expected to be present in both hydrogel phases, expanding them by increasing the D_2_O content. The decrease of |Δ*ρ*| and increase of *v*_p_ and *Q*_INV_ (see Tables 1 and 2) confirm this idea. The average radius of the spherical domains (⟨*R*⟩ = 41.2 Å), its polydispersity (*σ* = 0.149), and the effective radius of the HS interaction (*R*_eff_ = 53.4 Å) determined by fitting yield similar values as in the C_14_TAB subcritical hydrogels.

For the supercritical SDS hydrogels, the shapes of the scattering peaks are significantly different from the peaks registered for the subcritical SDS hydrogels. The peaks are similar to those observed in supercritical micellar SDS solutions; see, e.g., ref (63). Therefore, in these hydrogels, a new two-phase structure consisting of the SDS micelles dispersed in the highly swollen epoxy network (matrix phase) is expected. The high |Δ*ρ*|-values obtained by fitting confirm this idea since the scattering length density of the highly swollen phase approaches the value for heavy water (*ρ* = 6.35 × 10^10^ cm^–2^) and the scattering length density of the micellar core is formed by alkyl tails (*ρ* = −0.37 × 10^10^ cm^–2^); see data in Table 3. The average micelle radius remains constant at ca. 17 Å and is somewhat smaller than that found in supercritical SDS solutions (cross-sectional radius is ca. 20 Å SDS, based on data in ref (63)). The volume fractions of micelles are close to those of SDS molecules determined gravimetrically (see Table 1); therefore, a large part of SDS molecules is aggregated in the micelles. The micelles consist almost exclusively of SDS molecules. The average charge of micelles slightly increases from 7 to 12 elemental charges. This value is proportionally smaller than the value determined for SDS micelles in the supercritical SDS solutions (*q*_s_ ≈ 13 elemental charges).^63^

**Figure 11:**
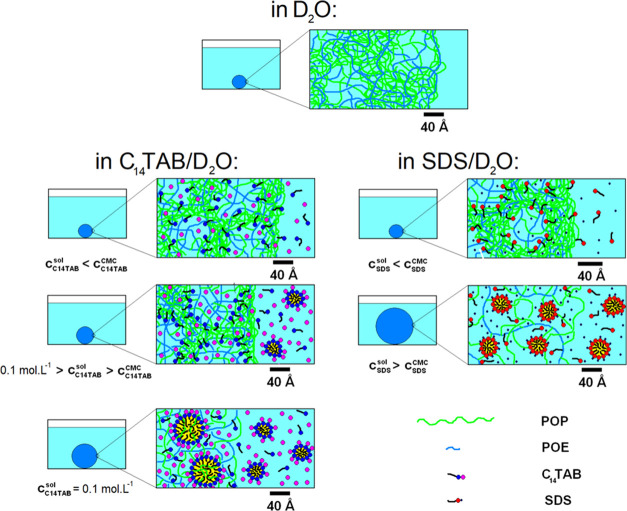
Schematic illustration of the effect of surfactant concentration in the swelling solution on hydrogel structure. Micelles with cross-sectional radii of ca. 17 and 20 Å found in supercritical C_14_TAB and SDS solutions, respectively, are depicted using numerical data from refs (71) and (63).

The structures of the hydrogels discussed above, which are consistent with the conclusions drawn from the SANS data, are illustrated schematically in Figure 11.

## Conclusions

A stoichiometric amphiphilic epoxy network containing POE and POP was prepared by the end-linking reaction of α,ω-diamino-terminated POP and POE bis(glycidyl ether) with molar masses of ca. 4000 and 526 g·mol^–1^, respectively. A series of hydrogels were prepared by swelling the network in D_2_O and subcritical and supercritical solutions of C_14_TAB and SDS in D_2_O.

The hydrogel obtained by swelling the network in neat D_2_O contains about 28 wt % D_2_O. Analysis of the SANS profile proves a two-phase nanophase-separated hydrogel structure consisting of polydisperse spherical domains with an average radius of ca. 39 Å (particulate phase) dispersed in a matrix and interacting via an extended HS potential. The particulate and matrix phases consist of highly and poorly swollen epoxy networks, respectively. The particulate phase contains about 44 vol % of the total D_2_O and about 1 vol % of the total epoxy network.

The presence of ionic surfactants (C_14_TAB and SDS) in the swelling solution had a strong effect on the swelling degree of the epoxy network and the structure of the resulting hydrogels.

In the subcritical region, the effects of both surfactants are similar. With increasing surfactant concentrations,

(i) At the macroscopic level, the equilibrium swelling ratio and surfactant uptake by the polymer network increase progressively. The concentrations of both surfactants in the hydrogels are much higher than those in the surrounding swelling solutions.
(ii) At the microscopic level, the two-phase nanophase-separated structure found in neat D_2_O is conserved; however, the size and composition of the phases are influenced by the presence of the surfactant. The surfactant molecules remain fully dissociated, hydrophobic alkyl tails are mixed with POP in the matrix phase, charged polar groups are located at the interface boundaries, and counterions are dispersed in both hydrogel phases. The radius of the spherical domains (⟨*R*⟩ = 41–45 Å) and their volume fraction (*v*_p_ = 0.2–0.3) increase with increasing surfactant concentration.

In the supercritical region, the effects of C_14_TAB and SDS differ significantly. With increasing surfactant concentration in the swelling solution:

(i) At the macroscopic level, the swelling degree increases for both surfactants, but much more strongly for SDS than for C_14_TAB. This is attributed to the smaller size and higher mobility of the sodium countercations relative to the bromine counteranions. The concentration of both surfactants in hydrogels remains much higher than their concentration in the surrounding swelling solution; however, their growth is less pronounced than in the subcritical region.
(ii) At the microscopic level, the two-phase nanophase-separated structure is preserved in the C_14_TAB hydrogels but not in the SDS ones. Only at the highest C_14_TAB concentration (0.1 mol·L^–1^), the poorly swollen matrix phase is disintegrated, and a new two-phase structure consisting of C_14_TAB micelles is dispersed in the highly swollen epoxy network matrix containing the remaining dissociated C_14_TAB molecules is formed. The micelles are larger (average radius is ca. 27 Å) than those formed in the surrounding swelling solution (average radius is ca. 17 Å), which is attributed to the strong binding of POP chains and alkyl tails in the micellar cores. There are about 2.3 elemental charges present on the micellar surface. In contrast, the micelles with an average radius of ca. 17 Å are formed in all SDS hydrogels. The water content in the highly swollen matrix phase is very high. The micelles are somewhat smaller than those in the surrounding swelling solution (average cross-sectional radius is ca. 20 Å) and have a proportionally smaller surface charge. Part of the dissociated nonaggregated SDS molecules remains in the highly swollen matrix phase.

The strong binding of the alkyl surfactant tails with POP chains and the dragging of a large amount of highly mobile counterions formed by the dissociation of the surfactants in an aqueous environment are responsible for the effects observed.

## Supporting Information Available

Epoxy network topology, polyoxypropylene chain dimensions, coherent small-angle neutron scattering profiles, and Porod fits (PDF)
