# Supplementary material for: Investigation of the Interaction of Ionic Surfactants with Epoxy-Based Hydrogels by SANS
Source: Langmuir. 2025 Feb 6;41(6):3895–908. doi: 10.1021/acs.langmuir.4c04062 (PMC11841043; doi:10.1021/acs.langmuir.4c04062)
Supplement: Supplementary file 1 — la4c04062_si_001.pdf [file la4c04062_si_001.pdf]

Supporting Information

Investigation of the Interaction of Ionic Surfactants with Epoxy-Based Hydrogels by SANS

Ivan Krakovský[*a], *Timur V. Tropin*[b,c], *Oleksandr Igorovych Ivankov*[b], *Viktor Petrenko*[c]

a) Department of Macromolecular Physics, Faculty of Mathematics and Physics, Charles University, V Holešovičkách 2, 180 00 Czech Republic

b) Frank Laboratory of Neutron Physics, Joint Institute for Nuclear Research, Joliot-Curie 6, 141980 Dubna, Russia

c) Basque Center for Materials, Applications & Nanostructures, Bld. Martina Casiano, UPV/EHU Science Park, Barrio Sarriena s/n, 48940 Leioa, Spain

* Corresponding author (e-mail: ivank@kmf.troja.mff.cuni.cz)

Contents of Supporting Information:

Number of pages: 5

Number of figures: 4

Table of Contents:

Figure S1. **a)** Topology of the stoichiometric epoxy network prepared by reaction of diamino terminated POP and diepoxy terminated POE, b) POP chain in stretched conformation and c) POP chain as a random coil.

S2. Estimation of the root of mean squared end-to-end distance of POP chain.

Figure S3. Log–log plot of coherent part of SANS profiles obtained from the epoxy network swollen to equilibrium in the $C_{14}TAB/D_2O$ solutions at 25 °C.

Figure S4. Log–log plot of coherent part of SANS profiles obtained from the epoxy network swollen to equilibrium in the SDS/D_2O solutions at 25 °C.

Figure S5. Porod fits in high-*q* region.

References

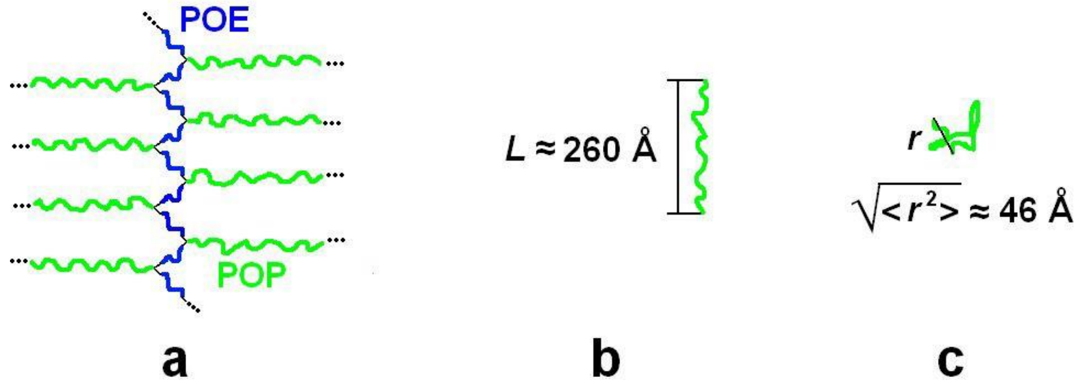

Figure S1. **a)** Topology of the stoichiometric epoxy network prepared by reaction of diamino terminated POP and diepoxy terminated POE, b) POP chain in a stretched conformation and c) POP chain in one of many random coil conformations.

S2. Estimation of the root of mean squared end-to-end distance of POP chain

The mean squared end-to-end distance of a sufficiently long POP chain under θ-conditions (in bulk or in θ-solvent) can be estimated by[S1]

$$\langle r^2 \rangle = C_\infty \left(n_{\mathrm{C-C}} l^2_{\mathrm{C-C}} + n_{\mathrm{C-O}} l^2_{\mathrm{C-O}} \right) \tag{S1}$$

where C_∞ is the characteristic ratio of POP, $n_{\mathrm{C\text{-}C}}$ ($n_{\mathrm{C\text{-}O}}$) and $l_{\mathrm{C\text{-}C}}$ ($l_{\mathrm{C\text{-}O}}$) the number of C-C (C-O) bonds in the POP chain backbone and the length of C-C (C-O) bond.

The numbers of bonds are related to the molar masses of POP chain, M_{POP}, and oxypropylene monomer unit, M_{OP}, by

$$n_{\mathrm{C-C}} = \frac{M_{\mathrm{POP}}}{M_{\mathrm{OP}}} \tag{S2}$$

$$n_{\mathrm{C-O}} = 2\frac{M_{\mathrm{POP}}}{M_{\mathrm{OP}}} \tag{S3}$$

By using numerical values M_{POP} = 4,000 g.mol^{-1}, M_{OP} = 58 g.mol^{-1}, $C_\infty \approx 5.05$ (POP in benzene[S1]), $l_{\mathrm{C\text{-}C}}$ = 1.5 Å and $l_{\mathrm{C\text{-}O}}$ = 1.4 Å in equations (S1) – (S3) one obtains the value of the root of the mean squared end-to-end distance $<r^2>^{1/2} \approx 46$ Å.

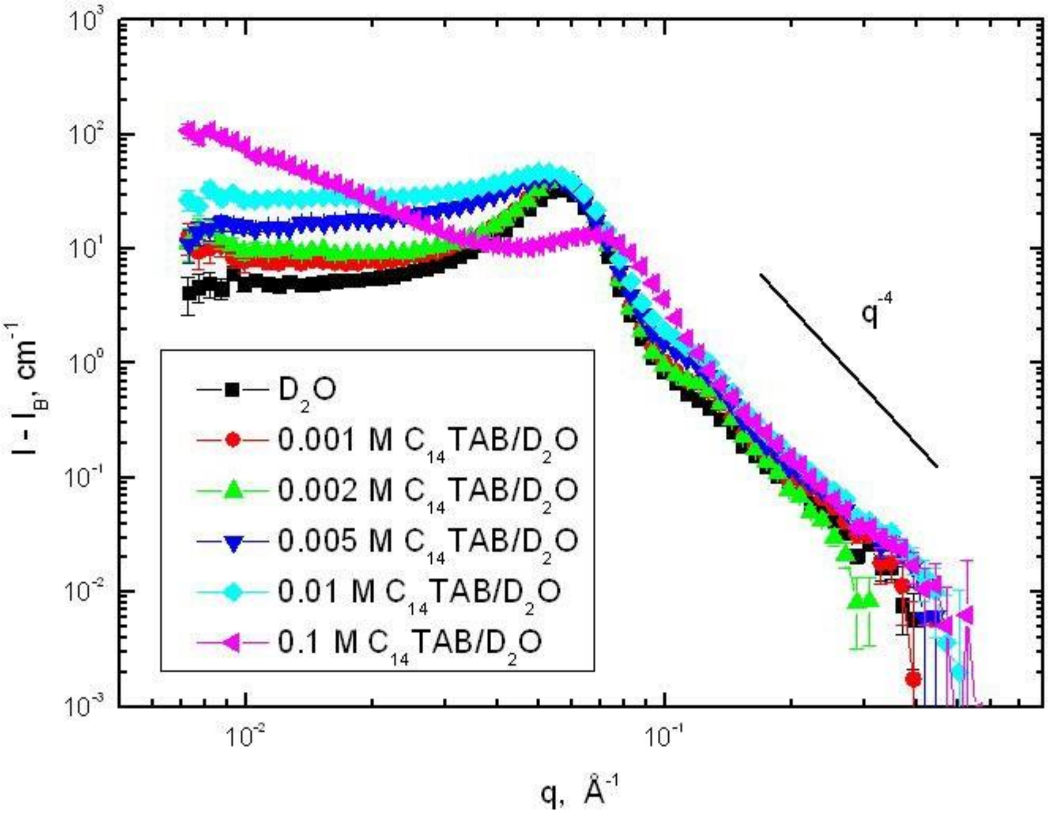

Figure S3. Log–log plot of coherent part of SANS profiles obtained from the epoxy network swollen to equilibrium in the $C_{14}TAB/D_2O$ solutions at 25 °C.

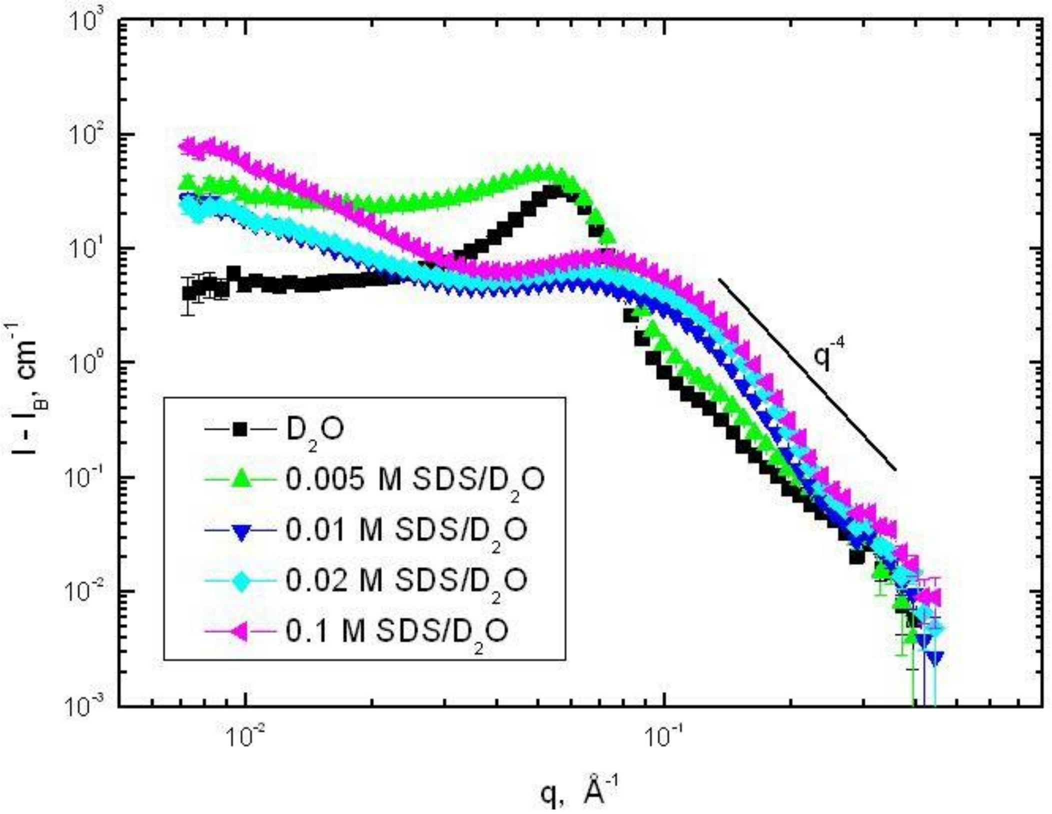

Figure S4. Log–log plot of coherent part of SANS profiles obtained from the epoxy network swollen to equilibrium in the SDS/D_2O solutions at 25 °C.

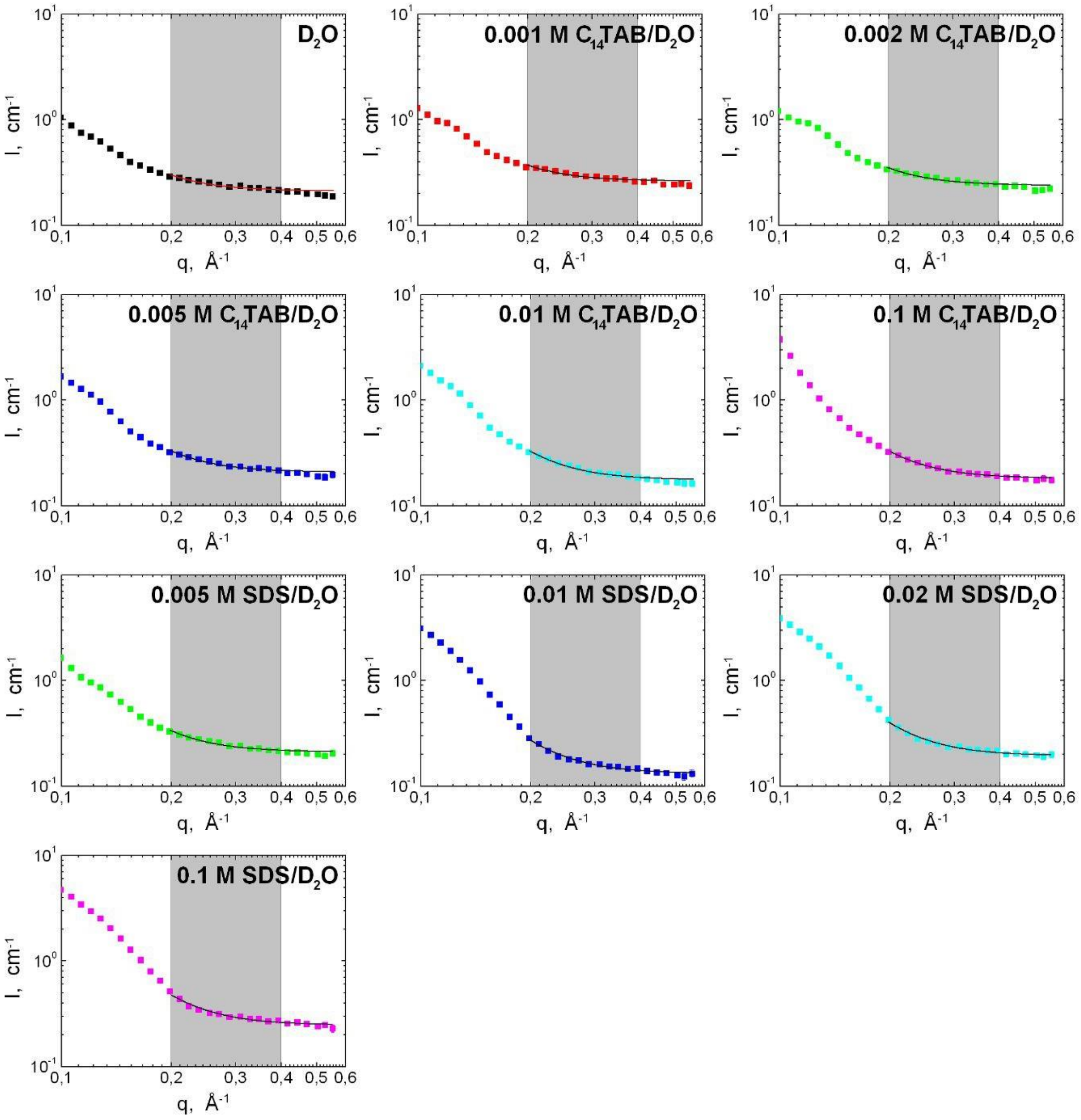

Figure S5. Porod fits in high-q region. Data at q > 0.4 Å^{-1} were excluded from the Porod fitting.

References

[S1] Brandrup, J.; Immergut, E. H.; Grulke, E. A. (Eds.). *Polymer Handbook (4th Ed.)*, Wiley-Blackwell 1999.
